# App-based conversational agents for tinnitus distress and mental health: A randomised controlled trial

**DOI:** 10.1016/j.invent.2026.100944

**Published:** 2026-04-12

**Authors:** James G. Jackson, Fayme Yeates, Ben Morris, Sophie Hollands, Grace E. Drybrough

**Affiliations:** School of Social Sciences, Leeds Trinity University, Horsforth, Leeds, LS18 5HD, United Kingdom

**Keywords:** Tinnitus, iCBT, Chatbot, Digital health, Mental health, Anxiety, Depression

## Abstract

Tinnitus is often accompanied by anxiety, depression, insomnia, and significant psychological distress, highlighting the need for accessible, evidence-based interventions. Internet-delivered cognitive behavioural therapy (iCBT) offers a scalable and standardised approach, and chatbots provide an emerging mode of automated delivery. This randomised controlled trial investigated the effectiveness of two app-based iCBT interventions for adults with persistent tinnitus (N = 105). Participants were randomly allocated to one of three conditions for eight weeks: a tinnitus-specific iCBT chatbot (Tinnibot), a general iCBT chatbot for mood and anxiety (Woebot), or a waiting-list control group. Outcomes were assessed at baseline and post-intervention and included tinnitus-related distress (Tinnitus Functional Index; primary outcome), anxiety (Generalised Anxiety Disorder Scale), depression (Patient Health Questionnaire), insomnia (Insomnia Severity Index), mindfulness (Mindful Attention Awareness Scale), and life satisfaction (Satisfaction with Life Scale). Analyses used mixed analysis of variance with an intention-to-treat approach. Both chatbot groups showed significant improvements in anxiety and depression relative to the control group. Tinnibot also produced significant improvements in insomnia severity. Tinnitus distress decreased in both chatbot groups, but only Tinnibot achieved a clinically meaningful reduction. Changes in mindfulness and life satisfaction were modest and did not differ between groups. These findings indicate that chatbot-delivered iCBT can improve comorbid symptoms associated with tinnitus, and that tinnitus-specific tailoring confers additional, clinically meaningful benefits for tinnitus distress.

## Highlights

• Both chatbots improved anxiety and depression when compared to control group.
• Tinnibot achieved greater tinnitus distress reductions than Woebot.
• Tinnibot also significantly reduced insomnia severity.
• Mindfulness and life satisfaction changes were modest across groups.
• Tinnitus-specific chatbot intervention showed additional benefits in this study.

## 1 Introduction

Tinnitus, the perception of sound in the absence of external stimuli, is a surprisingly common disorder, although prevalence estimates differ widely due to differences in study definitions and methodologies (McCormack et al., 2016). A large population-based study (n = 6098) by Oosterloo et al. (2021) suggests that 21.4% of the population has tinnitus. Up to one-third of individuals with tinnitus are estimated to have substantial distress, with 1–2% considering the condition to be profoundly disabling (Langguth et al., 2013). In a recent systematic review and meta-analysis, Jiang et al. (2025) reported that tinnitus is significantly associated with anxiety, depression, insomnia and suicidal ideation. The effect of chronic tinnitus on mental health is profound and in the case of the most significantly affected, effective interventions are urgently needed.

Tinnitus-related distress does not have a single cause and is most likely shaped by a combination of factors. While some have proposed that tinnitus distress is moderated by negative cognitions (Jackson and Woolmer, 2024) or individual differences (Biehl et al., 2020), it is clear that tinnitus distress does not arise from the sensation itself. Instead, it is suggested that the interaction and maintenance between cognitions, emotions, attention and behaviours regarding tinnitus contribute to the psychological distress experienced (Handscomb et al., 2020).

Treatments based on cognitive behavioural therapy (CBT) principles aim to target these processes, focusing on emotional coping and perceptual habituation rather than direct suppression of the tinnitus sound (Fuller et al., 2020). CBT interventions for tinnitus can significantly improve quality of life, reduce tinnitus distress, and alleviate comorbid anxiety, depression, and insomnia (Zhong et al., 2025). However, delivery in clinical practice is inconsistent. In the UK, National Institute for Health and Care Excellence (NICE) guidelines recommend psychological input for persistent distress (National Institute for Health and Care Excellence [NICE], 2020). In practice this may be provided by psychologists, accredited CBT therapists, audiologists with some CBT training, or hearing healthcare professionals without any formal CBT credentials. This variation leads to uneven quality and a lack of standardisation across services.

Digital delivery offers a potential solution to these disparities, allowing for consistent, standardised CBT content that is not dependent on the training background or availability of individual clinicians. Recent efforts have adapted CBT for internet delivery (iCBT), offering scalable and cost-effective access to care (Beukes et al., 2018). In both therapist-guided and self-help forms, iCBT shows effects comparable to face-to-face delivery (Fuller et al., 2020), though implementation can vary considerably across programmes. Still, the model offers the potential for more uniform delivery of core CBT components. Building on this, app-based conversational agents (i.e. chatbots) represent a further evolution in iCBT. Smartphone delivery chatbots can offer enhanced accessibility, user engagement, and the ability to deliver highly standardised, evidence-based content at scale. Evidence supporting the efficacy of chatbot-based iCBT for depression and anxiety spans early work such as Fitzpatrick et al. (2017) demonstrating the benefits of the Woebot platform for depressive symptoms, and more recent large-scale evaluations confirming effectiveness across diverse populations (He et al., 2023). However, research into their potential for tinnitus patients remains limited. Bardy et al. (2024) suggested possible benefits in reducing distress, but the study was small (n = 28) and lacked a control condition.

### 1.1 Research aims and hypotheses

The present study aimed to address this gap through a randomised controlled trial comparing participants across three conditions: Tinnibot (a tinnitus-specific iCBT application), Woebot (a general iCBT application for anxiety/depression), or a waiting list control. By including both a condition-specific and a generic smartphone intervention, we hypothesised that (i) app-based iCBT should be effective in reducing tinnitus-related distress, and (ii) that tinnitus-specific applications confer additional benefits for tinnitus patients.

## 2 Method

This was a three-arm, parallel-group, open-label randomised controlled trial conducted over an eight-week intervention period.

### 2.1 Participants

108 adult participants experiencing persistent tinnitus volunteered, of which 105 eventually participated (age range: 22–86, M = 53.76 yrs., SD = 12.75). 54 were male and 51 were female. Participants reported a mean tinnitus duration of 129.85 months (SD = 93.07), indicating a predominantly chronic tinnitus population. Eligibility included: (i) being aged eighteen years or older; (ii) self-reported persistent tinnitus for at least three months; (iii) fluency in English; and (iv) access to a smartphone. Eligibility screening was based on self-reported inclusion and exclusion criteria only and did not involve formal medical, audiological, or hearing assessments. Participants were recruited via online advertisements and with support from Tinnitus UK. Participants were excluded if they were currently engaged in other treatments and/or interventions for tinnitus. Interested individuals contacted the research team directly to express interest in participation before being randomly allocated to one of three conditions. Participants were then granted access to an online survey page that provided study information as well as an opportunity to confirm informed consent. Compensation included a payment of £10 on completion of data collection and a free subscription to the Tinnibot smartphone application. Experimental protocols were approved by **[REDACTED FOR ANONYMITY]** and conform with ethical standards laid down in the 1964 Declaration of Helsinki.

### 2.2 Design

A randomised controlled trial design was adopted since this constitutes the ‘gold standard’ approach of investigating intervention outcomes (McCoy, 2017). By way of a pre-set randomisation programme (Research Randomizer, e.g. Jackson and Woolmer, 2024), participants were randomly allocated to one of three conditions: Woebot (n = 36), Tinnibot (n = 36), or Waiting List Control (n = 36). Three participants later withdrew, one from each condition. The primary outcomes were self-reported symptoms of anxiety, depression, insomnia, mindfulness, life satisfaction, and tinnitus-related distress. These outcomes were measured at baseline and at the end of an eight-week intervention. Therefore, this study used a 2 × 3 mixed ANOVA design. Condition (Waiting List Control, Woebot, Tinnibot) was the between-subjects factor and Time (Baseline, Post-intervention) was the within-subjects factor. Analyses followed an intention-to-treat (ITT) approach, with all randomised participants analysed in their assigned groups. Missing data were inputted using the Last Observation Carried Forward (LOCF) method.

### 2.3 Procedure

After eligibility screening and informed consent, participants completed self-reported baseline assessments through an online survey platform (www.onlinesurveys.ac.uk). As the study was designed as a remote online trial to maximise accessibility and permit participation across a geographically dispersed sample, no clinical interview, audiological examination, or formal hearing assessment was conducted as part of screening or outcome assessment. The trial was open label, meaning that participants were aware of their assigned condition, and outcome assessment relied on self-reported measures collected online. Participants in the Woebot and Tinnibot groups also received weekly instructions to engage with their assigned digital intervention over an eight-week period. Participants were reassessed after eight weeks using the same online platform. The waitlist control group received no intervention, but all participants were offered access to remaining digital support tools upon completion.

At baseline, all participants were asked to provide demographic details including self-reported extent of hearing difficulties by way of the Hearing Handicap Inventory for Adults (HHI-A). At baseline and again post-intervention, participants completed self-report scales for Anxiety (General Anxiety Disorder Scale, GAD-7), Depression (Patient Health Questionnaire, PHQ-9), Insomnia symptoms (Insomnia Severity Index, ISI), Mindfulness (Mindfulness Awareness Scale, MAAS-15), Satisfaction with Life (Satisfaction with Life Scale, SWLS), and Tinnitus Distress (Tinnitus Functional Index, TFI).

After the baseline survey was completed, Tinnibot and Woebot participants were provided with instructions on how to download their designated smartphone application. On confirmation that they had been successful, participants were given eight weeks before completing their second online survey. Smartphone users were asked to engage with their assigned App at least once per day, although engagement was self-paced and not actively monitored. Weekly email reminders were sent to encourage continued use. After completion, participants were reimbursed for their time. If they wished, this was offered to charitable causes instead. All participants were then debriefed and provided the ability to access remaining interventions in their own time.

### 2.4 Interventions

#### 2.4.1 Tinnibot

Tinnibot was a tinnitus-specific digital health intervention delivered via a smartphone application during the data collection period. Unlike general mental health chatbots, Tinnibot focused exclusively on tinnitus-related distress, integrating internet-based cognitive behavioural therapy (iCBT) with mindfulness-based cognitive therapy (MBCT) principles (Philippot et al., 2012; Makar et al., 2017). Its core features included tailored psychoeducation, cognitive restructuring, mindfulness-guided meditation, and sound therapy modules designed specifically for tinnitus symptom management.

The application employed a conversational agent (“chatbot”) to simulate a therapeutic dialogue, offering asynchronous, user-paced interactions that sought to replicate supportive clinical consultations. This tinnitus-specific tailoring distinguished Tinnibot from broader mental health chatbots by addressing the unique psychological and perceptual challenges associated with chronic tinnitus. Empirical support for Tinnibot's efficacy comes from a recent randomised controlled trial by Bardy et al. (2024), which demonstrated significant reductions in tinnitus-related distress, depression, and anxiety after an eight-week intervention. Clinically meaningful improvements were observed in 42% of participants using Tinnibot alone, rising to 64% among those receiving additional telepsychological support, underscoring its value as both a standalone and hybrid treatment.

While Tinnibot has since been rebranded and relaunched as Mindear (Tinnibot version 2.0), the present study utilised the original version available at the time, offering critical insight into the role of domain-specific digital therapeutics in tinnitus care.

#### 2.4.2 Woebot

Woebot was a general-purpose, AI-driven mental health chatbot designed to deliver evidence-based cognitive behavioural therapy (CBT) through brief, structured, and empathic conversations. Available as a smartphone App, it supported daily engagement and offered tools for mood tracking, cognitive distortion identification, cognitive reframing, and access to short psychoeducational content. Interactions were guided by a conversational agent (“Woebot”) that used natural language processing to simulate supportive and adaptive dialogue. Key features included mood journaling, mindfulness exercises, interactive videos, and just-in-time interventions targeting distressing thought patterns (Fitzpatrick et al., 2017).

Participants were encouraged to engage with Woebot daily over the eight-week intervention period, with most sessions lasting 5–10 min. Each session typically began with a mood check-in, followed by a guided CBT-based conversation and, where appropriate, the delivery of brief multimedia content such as mindfulness prompts or psychoeducational videos. The experience was fully automated and asynchronous, allowing users to access support at their convenience.

Woebot has demonstrated efficacy in reducing symptoms of anxiety, depression, stress, and burnout in both clinical and non-clinical populations (Durden et al., 2023; Prochaska et al., 2021). It incorporated automated triage and escalation protocols to prioritise user safety, including referral prompts when users disclosed risk. Although the App was withdrawn from public use in June 2025, it remains a prominent example of relational agent-based digital mental health intervention. Participants in the present study accessed Woebot prior to its closure.

### 2.5 Measures

#### 2.5.1 Generalized Anxiety Disorder Scale (GAD-7)

The GAD-7 (Spitzer et al., 2006) is a succinct 7-item self-report questionnaire designed to screen for symptoms of generalized anxiety disorder. Respondents rate how often they have been bothered by anxiety-related experiences (e.g., “Feeling nervous, anxious or on edge” or “Not being able to stop or control worrying”) using a 4-point Likert scale from 0 (“not at all”) to 3 (“nearly every day”). Total scores range from 0 to 21, with higher scores reflecting greater anxiety symptom severity.

Interpretative guidelines suggest cut-offs of 5, 10, and 15 to indicate mild, moderate, and severe anxiety respectively (Spitzer et al., 2006). The GAD-7 has demonstrated strong psychometric properties in populations with tinnitus distress and symptoms of anxiety (Trevis et al., 2018). In the current study, the GAD-7 demonstrated good internal consistency at baseline (ω = 0.89) and post-intervention (ω = 0.86).

#### 2.5.2 Hearing Handicap Inventory for Adults (HHI-A)

The Hearing Handicap Inventory for Adults (Newman et al., 1990) is a 25-item self-report measure designed to assess the perceived social and emotional impact of hearing difficulties. Items are rated on a three-point scale: “Yes” (4 points), “Sometimes” (2 points), and “No” (0 points), yielding a total score ranging from 0 to 100. Higher scores indicate greater self-perceived hearing handicap. Example items include “Does a hearing problem cause you to feel frustrated when talking to family members?” and “Does a hearing problem cause you to avoid social gatherings?”

The HHI-A has demonstrated strong internal consistency and construct validity in both audiological and general health contexts (Cassarly et al., 2020) and has been widely used in tinnitus research to assess the broader psychosocial burden of auditory symptoms. The HHI-A was completed at baseline only and was treated as a descriptive measure of hearing difficulty rather than a longitudinal outcome. It demonstrated good internal consistency (ω = 0.96).

#### 2.5.3 Insomnia Severity Index (ISI)

The Insomnia Severity Index (Bastien et al., 2001) is a widely used 7-item self-report scale assessing the nature, severity, and impact of insomnia symptoms. Items address difficulty with sleep onset, sleep maintenance, early morning awakenings, satisfaction with sleep patterns, interference with daily functioning, and associated distress. Responses are rated on a 5-point Likert scale (0–4), with total scores ranging from 0 to 28. Higher scores reflect more severe insomnia symptoms.

Interpretative guidelines (Morin et al., 2011) suggest cut-off scores of 0–7 (no clinically significant insomnia), 8–14 (subthreshold insomnia), 15–21 (moderate severity), and 22–28 (severe insomnia). The ISI has demonstrated strong internal consistency and test–retest reliability in both general and clinical populations (e.g., Castronovo et al., 2016). In the present study, the ISI demonstrated good internal consistency at baseline (ω = 0.94) and post-intervention (ω = 0.92).

#### 2.5.4 Mindful Attention Awareness Scale (MAAS-15)

The Mindful Attention Awareness Scale (Brown and Ryan, 2003) is a 15-item self-report instrument that assesses a core component of mindfulness: the capacity for present-moment attentiveness and awareness. Respondents rate how frequently they experience lapses in attention or operate on “autopilot” using a 6-point Likert scale (1 = almost always, 6 = almost never). Example items include “I could be experiencing some emotion and not be conscious of it until sometime later” and “I find myself doing things without paying attention.” All items are negatively phrased, so higher total scores (range: 15–90) reflect greater dispositional mindfulness in the form of sustained attention and awareness.

Although the MAAS-15 captures a unidimensional construct—specifically attentional awareness—it does not address other facets of mindfulness, such as nonjudgmental acceptance. Nevertheless, it remains a psychometrically robust and widely used measure, particularly suitable for contexts in which attentional processes are central. Given the relevance of attentional focus in the perception and appraisal of tinnitus, the MAAS-15 was deemed appropriate for the current study's focus on tinnitus monitoring and related psychological outcomes.

The scale has demonstrated strong internal consistency and a stable unidimensional factor structure across diverse populations (e.g., Hyland et al., 2015; MacKillop and Anderson, 2007). Its scores have also been linked to constructs such as subjective well-being, life satisfaction, and self-compassion (González-Blanch et al., 2022). In the present study, the MAAS-15 demonstrated good internal consistency at baseline (ω = 0.94) and post-intervention (ω = 0.93).

#### 2.5.5 Patient Health Questionnaire (PHQ-9)

The PHQ-9 (Kroenke et al., 2001) is a nine-item self-report measure designed to assess the severity of depressive symptoms over the previous two weeks. Respondents rate each item on a four-point Likert scale (0 = not at all to 3 = nearly every day), yielding a total score ranging from 0 to 27, with higher scores indicating greater depression severity. Example items include “Little interest or pleasure in doing things” and “Feeling down, depressed, or hopeless.”

Established interpretative cut-offs define scores of 5, 10, 15, and 20 as indicative of mild, moderate, moderately severe, and severe depression respectively (Kroenke et al., 2001). The PHQ-9 is one of the most widely used depression screening tools globally and has demonstrated strong psychometric properties in both clinical and community samples. In their very recent development of the Tinnitus Qualities and Impact Questionnaire, Manchaiah et al. (2025) report good Cronbach's α values (α = 0.90) for the PHQ-9 in a tinnitus population (n = 660). In the present study, the PHQ-9 demonstrated good internal consistency at baseline (ω = 0.89) and post-intervention (ω = 0.88).

#### 2.5.6 Satisfaction with Life Scale (SWLS)

The Satisfaction with Life Scale (Diener et al., 1985) is a 5-item instrument designed to measure global cognitive judgments of life satisfaction. Participants rate items such as “In most ways my life is close to my ideal” and “I am satisfied with my life” on a 7-point Likert scale ranging from 1 (“strongly disagree”) to 7 (“strongly agree”). Total scores range from 5 to 35, with higher scores indicating greater satisfaction with life.

The SWLS has consistently demonstrated strong psychometric properties, including a unidimensional factor structure, high internal consistency, and good convergent validity with other indicators of well-being (Pavot and Diener, 2008). It has been widely used in health psychology research, including studies exploring tinnitus-related distress. In the present study, the SWLS demonstrated good internal consistency at baseline (ω = 0.93) and post-intervention (ω = 0.93).

#### 2.5.7 Tinnitus Functional Index (TFI)

The TFI (Meikle et al., 2012) is a 25-item self-report measure which quantifies the functional impact of tinnitus across multiple life domains, including emotional well-being, concentration, sleep, and auditory interference. Respondents rate items on an 11-point scale (0−10), with questions such as “How difficult was it to cope with your tinnitus?” and “How much has your tinnitus interfered with your ability to enjoy peace and quiet?” Total raw scores are converted to a standardised scale from 0 to 100, with higher values indicating greater severity of impact.

Interpretative cut-offs proposed by Henry et al. (2016) suggest that scores of 0–24 reflect minimal need for intervention, 25–49 indicate moderate functional difficulties, and scores of 50 or above denote severe tinnitus-related distress requiring clinical attention. The TFI has seen widespread international adoption in both clinical and research settings, supported by multiple language translations and robust psychometric properties. In a UK-based sample, Fackrell et al. (2016) reported excellent internal consistency (α = 0.90), and in a separate study using the same scale, Jackson and Woolmer (2024) reported comparable reliability using McDonald's omega (ω = 0.95). In the present study, the TFI demonstrated excellent internal consistency at baseline (ω = 0.97) and post-intervention (ω = 0.97).

### 2.6 Statistical analysis

All analyses were conducted in SPSS (Version 29). Six 2 × 3 mixed ANOVAs tested for Time × Condition interactions across outcomes. Paired-sample *t*-tests were used to explore within-group changes over time. Effect sizes are reported as partial eta squared (η^2^ₚ). Assumptions of normality and homogeneity of variance were assessed and met.

## 3 Results

To evaluate our hypotheses regarding the impact of two smartphone-delivered mental health interventions (Woebot and Tinnibot) compared to a waiting list control, we conducted a series of 2 (Time: Pre-/Post-Intervention) × 3 (Condition: Control, Woebot, Tinnibot) mixed ANOVAs. Outcomes included anxiety (GAD-7), depression (PHQ-9), insomnia (ISI), mindfulness (MAAS-15), life satisfaction (SWLS), and tinnitus-related distress (TFI). For each outcome, we report main effects of Time and Condition, as well as the critical Time × Condition interaction. Where significant interactions emerged, we conducted follow-up paired-sample t-tests and simple effects analyses. Descriptive statistics appear in Table One.

Prior to outcome analysis, a univariate ANOVA confirmed no significant baseline differences in self-reported hearing handicap (HHI-A) across groups [F(2,102) = 0.74, *p* = .481, η^2^ₚ = 0.014] (Table 1).

**Table 1:** Estimated marginal means (standard errors), mean differences, and effect sizes by dependent variable and condition.

Dependent variable	Condition	Baseline mean (SE)	Post-intervention mean (SE)	Mean change	p	[95% CI]	η^2^ₚ
Hearing Handicap (HHIA)	Control	32.23 (3.97)	–	–	–	–	–
	Woebot	31.65 (4.08)	–	–	–	–	–
	Tinnibot	38.33 (4.16)	–	–	–	–	–
Anxiety (GAD-7)	Control	8.46 (0.47)	9.17 (0.48)	0.71	.254 ns.	[−1.96, 0.54]	0.04
	Woebot	8.82 (0.52)	7.35 (0.47)	−1.47	0.022	[0.22, 2.72]	0.04
	Tinnibot	10.94 (0.58)	8.44 (0.47)	−2.50	<0.001	[1.34, 3.66]	0.10
Depression (PHQ-9)	Control	9.29 (0.49)	9.64 (0.50)	0.35	.511 ns.	[−1.43, 0.73]	0.01
	Woebot	10.03 (0.53)	8.06 (0.48)	−1.97	0.005	[0.62, 3.32]	0.06
	Tinnibot	11.97 (0.60)	8.88 (0.49)	−3.09	<0.001	[1.76, 4.42]	0.11
Mindfulness (MAAS-15)	Control	4.11 (0.20)	4.08 (0.18)	−0.03	.455 ns.	[−0.34, 0.28]	<0.01
	Woebot	3.74 (0.17)	4.13 (0.17)	0.39	<0.001	[−0.01, 0.79]	0.08
	Tinnibot	3.71 (0.16)	3.95 (0.14)	0.24	0.006	[−0.12, 0.60]	0.04
Satisfaction with Life (SWLS)	Control	21.26 (1.19)	21.26 (1.34)	0.00	1.000	[−0.331, 0.331]	0.00
	Woebot	20.62 (1.27)	21.62 (1.23)	1.00	.660 ns.	[−0.606, 0.79]	0.07
	Tinnibot	19.75 (1.17)	21.42 (1.28)	1.67	0.047	[−0.618, 0.049]	0.08
Sleep Quality (ISI)	Control	11.83 (1.17)	12.29 (1.10)	0.46	.401 ns.	[−1.60, 2.52]	<0.01
	Woebot	11.50 (1.19)	10.65 (1.11)	−0.85	.123 ns.	[−0.25, 1.95]	<0.01
	Tinnibot	13.03 (1.16)	10.44 (1.08)	−2.59	0.002	[1.01, 4.17]	0.08
Tinnitus Distress (TFI)	Control	49.25 (3.33)	49.19 (3.52)	−0.06	.983 ns.	[−5.38, 5.49]	0.00
	Woebot	50.11 (3.38)	42.73 (3.57)	−7.38	0.009	[1.86, 12.89]	0.07
	Tinnibot	52.66 (3.28)	39.42 (3.47)	−13.23	<0.001	[7.87, 18.59]	0.20

Note. Estimated marginal means are based on mixed ANOVA analyses. η^2^ₚ = partial eta squared. CI = confidence interval. All comparisons reflect within-group changes from pre- to post-intervention.

### 3.1 Anxiety (GAD-7)

There was no significant main effect of Condition [F(2,102) = 1.02, *p* = .363, η^2^ₚ = 0.020], though there was a significant main effect of Time [F(1,102) = 9.12, *p* = .003, η^2^ₚ = 0.082], indicating a reduction in anxiety over time. The Time × Condition interaction was significant [F(2,102) = 7.02, *p* = .001, η^2^ₚ = 0.121]. At baseline, GAD-7 scores did not differ between groups (*p* > .05), confirming groups were comparable at baseline.

Pairwise comparisons indicated that the control group showed no significant change in anxiety scores after two months: M = 8.46 (SE = 0.47) at baseline and M = 9.17 (SE = 0.48) post-intervention; *mean difference* = +0.71, *p* = .254, 95% CI [−1.96, 0.54]. Partial eta squared is not reported here due to negligible effects. Participants using the Woebot smartphone application demonstrated a significant reduction in anxiety from M = 8.82 (SE = 0.52) to M = 7.35 (SE = 0.47); *mean difference* = −1.47, *p* = .022, 95% CI [0.22, 2.72], η^2^ₚ = 0.044. Tinnibot participants reported a larger reduction: M = 10.94 (SE = 0.58) at baseline and M = 8.44 (SE = 0.47) post-intervention; *mean difference* = −2.50, *p* < .001, 95% CI [1.34, 3.66], η^2^ₚ = 0.095.

### 3.2 Depression (PHQ-9)

There was no significant main effect of Condition on self-reported depression [F(2,102) = 0.50, *p* = .675, η^2^ₚ = 0.010]. A significant main effect of Time emerged, F(1,102) = 4.87, *p* = .030, η^2^ₚ = 0.046, with lower depression scores post-intervention. The Time × Condition interaction was also significant [F(2,102) = 3.43, *p* = .036, η^2^ₚ = 0.063]. Baseline PHQ-9 scores did not differ across groups (*p* > .05). Waiting List Controls showed no significant change in depression scores after two months: M = 9.29 (SE = 0.49) at baseline and M = 9.64 (SE = 0.50) post-intervention; *mean difference* = 0.35, *p* = .511, 95% CI [−1.43, 0.73]. Partial eta squared not reported here due to negligible effects. Participants using the Woebot smartphone application demonstrated a significant reduction in depression from M = 10.03 (SE = 0.53) to M = 8.06 (SE = 0.48); *mean difference* = −1.97, *p* = .005, 95% CI [0.62, 3.32], η^2^ₚ = 0.062. Tinnibot users reported a larger reduction: M = 11.97 (SE = 0.60) at baseline and M = 8.88 (SE = 0.49) post-intervention; *mean difference* = −3.09, p < .001, 95% CI [1.76, 4.42], η^2^ₚ = 0.112.

### 3.3 Insomnia Severity (ISI)

There was no main effect of Condition [F(2,102) = 0.23, *p* = .799, η^2^ₚ = 0.004] but a significant main effect of Time was found [F(1,102) = 3.92, *p* = .050, η^2^ₚ = 0.037]. A significant Time × Condition interaction emerged [F(2,102) = 3.13, *p* = .048, η^2^ₚ = 0.058]. At baseline, groups were equivalent (p > .05).Utilised pre-post comparisons showed distinct differences between groups. Waiting List Controls showed no significant change in insomnia severity scores after two months: M = 11.83 (SE = 1.17) at baseline and M = 12.29 (SE = 1.10) post-intervention; *mean difference* = −0.46, *p* = .401, 95% CI [−1.60, 2.52]. Partial eta squared not reported here due to negligible effects. There was a non-significant reduction for the Woebot condition: M = 11.50 (SE = 1.19) at baseline and M = 10.65 (SE = 1.11) post-intervention; *mean difference* = −0.85, *p* = .123, 95% CI [−0.25, 1.95], η^2^ₚ = 0.009. In contrast, users in the Tinnibot condition demonstrated a significant reduction in sleep disturbance: M = 13.03 (SE = 1.16) at baseline and M = 10.44 (SE = 1.08) post-intervention; *mean difference* = −2.59, *p* = .002, 95% CI [1.01, 4.17], η^2^ₚ = 0.082.

### 3.4 Mindfulness (MAAS-15)

There was no significant main effect of Condition on trait mindfulness [F(2,102) = 0.712, *p* = .493, η^2^ₚ = 0.014]. However there was a significant main effect of time [F(1,102) = 7.850, *p* = .006, η^2^ₚ = 0.071], demonstrating significant changes in trait mindfulness over the eight-week intervention period. The interaction between time and condition did not reach statistical significance [F(2,102) = 2.847, *p* = .063, η^2^ₚ = 0.053], indicating no strong evidence that changes in mindfulness over time differed by condition. Exploratory paired-sample *t*-tests showed that Woebot participants increased in mindfulness [t(38) = 3.64, *p* = .001], as did Tinnibot participants [t(38) = 2.88, p = .006], while the control group did not [t(37) = 0.77, *p* = .445]. Given the non-significant interaction, these within-group results are exploratory and not interpreted further. See Table One for descriptives.

### 3.5 Satisfaction with Life (SWLS)

A 2 × 3 mixed ANOVA revealed a significant main effect of Time [F(1,102) = 4.06, *p* = .047, η^2^ₚ = 0.038], with modest improvements in life satisfaction across conditions. There was no main effect of Condition [F(2,102) = 0.09, *p* = .913, η^2^ₚ = 0.002], and no significant Time × Condition interaction [F(2,102) = 1.22, *p* = .300, η^2^ₚ = 0.053]. In exploratory follow-ups, controls showed no change [t(34) = 0.00, *p* = 1.000]. Woebot participants showed a trend toward improvement [t(33) = −1.55, *p* = .066, one-tailed], and Tinnibot participants showed a statistically significant increase [t(35) = −1.72, p = .047, one-tailed], consistent with hypothesised effects Given the absence of a significant interaction. These results are not interpreted further. See Table One for descriptives.

### 3.6 Tinnitus Distress (TFI)

**Fig. 1:**
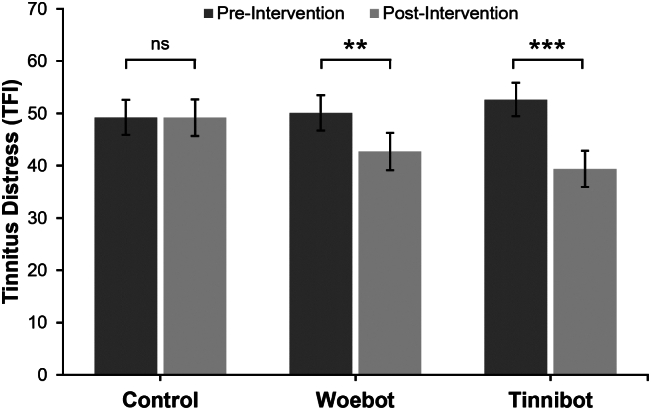
Mean Tinnitus Distress (TFI) comparing each condition pre- and post-intervention. *Note*. The overall Condition × Time interaction was significant (*p* < .01). ***p < .001; **p < .01; ns *p* > .05. Error bars represent standard error.

There was no significant main effect of Condition on tinnitus distress [F(2,102) = 0.307, *p* = .750, η^2^ₚ = 0.006], though there was a significant main effect of Time [F(1,102) = 18.947, *p* < .001, η^2^ₚ = 0.157] indicating reductions in tinnitus distress across eight weeks. There was also a significant interaction between Condition and Time [F(2,102) = 5.878, *p* = .004, η^2^ₚ = 0.103]. To explore this interaction, we examined estimated marginal means before conducting pairwise comparisons and simple effects analyses. At baseline TFI scores did not differ between three groups (*p* > .05), indicating successful randomisation and group equivalence. However, pre-post comparisons within each group revealed differential patterns of change over time. The Waiting List Control Group showed no significant change over time: *M* = 49.25 (SE = 3.33) at baseline and *M* = 49.19 (SE = 3.52) post-intervention; *mean difference* = −0.06, *p* = .983, 95% CI [−5.38, 5.49]. Partial eta squared not reported here due to negligible effects. Participants making use of the Woebot application demonstrated a significant reduction in tinnitus distress from *M* = 50.11 (SE = 3.38) to *M* = 42.73 (SE = 3.57); *mean difference* = −7.38, *p* = .009, 95% CI [1.86, 12.89], η^2^ₚ = 0.065. Tinnibot participants reported a larger and clinically meaningful reduction: *M* = 52.66 (SE = 3.28) at baseline and *M* = 39.42 (SE = 3.47) post-intervention; *mean difference* = −13.23, *p* < .001, 95% CI [7.87, 18.59], η^2^ₚ = 0.190 (Fig. 1).

### 3.7 Summary

In summary, both Woebot and Tinnibot produced improvements in anxiety and depression relative to the control group, though effects were stronger and more consistent for Tinnibot. Tinnibot also produced significant improvements in insomnia severity. Importantly, Tinnibot was the only intervention to produce clinically meaningful reductions in tinnitus-related distress. Changes in mindfulness and life satisfaction were modest and did not differ significantly between groups. The control group showed no reliable changes. These findings therefore provide partial support for our first hypothesis, that iCBT apps reduce tinnitus-related distress and comorbid symptoms, as well as stronger support for our second hypothesis, that tinnitus-specific interventions yield greater benefits than the generic CBT-based chatbot (Woebot) in this study.

## 4 Discussion

The present study provides novel evidence that smartphone-delivered conversational agents can reduce tinnitus-related distress and comorbid psychological symptoms. Both Woebot and Tinnibot were associated with improvements in anxiety, depression, and insomnia – but only Tinnibot produced clinically meaningful reductions in tinnitus-related distress. These findings extend previous evaluations of internet-based CBT (iCBT) and online tinnitus interventions (e.g. Sia et al., 2024) by demonstrating the feasibility and efficacy of fully automated, app-based delivery, while highlighting the added value of condition-specific tailoring for individuals with tinnitus.

The findings offer partial support for our first hypothesis, predicting that app-based iCBT would reduce tinnitus-related distress and associated comorbidities. Improvements were observed in comorbid symptoms across both chatbot conditions, but tinnitus distress was only meaningfully reduced in the tinnitus-specific intervention (Tinnibot). This pattern aligns with transdiagnostic models of CBT, which propose that shared mechanisms – e.g. worry, rumination, and attentional bias – underpin many forms of psychological distress. Comparable results were recently reported by Bardy et al. (2024) and Xian et al. (2025), both of whom demonstrated that internet-delivered CBT can reduce tinnitus distress while improving mood and sleep quality. In addition, Jackson and Woolmer (2024) demonstrated similar benefits using an online mindfulness-based intervention for tinnitus, further supporting the value of interventions that target general cognitive-emotional processes even when not explicitly tailored to tinnitus.

Our results offer support for the second hypothesis, that a tinnitus-specific application can confer additional benefits beyond the generic chatbot comparator used in this study. Participants using Tinnibot reported larger reductions in tinnitus-related distress, consistent with the view that it is perceptual and cognitive mechanisms which maintain tinnitus symptoms. Accordingly, tinnitus-specific psychoeducation, sound therapy, and mindfulness practices can more directly support the needs of tinnitus patients. This interpretation is supported by Jiang et al. (2025) who reported that CBT was particularly effective in reducing tinnitus comorbidities such as depression and insomnia, whereas the addition of sound therapy in parallel was increasingly effective in reducing tinnitus distress. Broader reviews of digital health interventions (e.g. Sattel et al., 2025) also highlight the importance of tailoring content to disorder-specific mechanisms when maximising treatment impact. Reviews of conversational agents (e.g. Casu et al., 2024) consistently demonstrate meaningful improvements in mental health outcomes, underscoring the viability of relational agents as delivery formats. However, user experience and engagement challenges remain, and the literature increasingly suggests that chatbots should be tailored for specific populations and contexts. This interpretation is supported by the stronger outcomes observed for Tinnibot relative to Woebot in the present study. Complementing this, Mayor's (2025) umbrella review highlights the rapid growth of chatbot research but also cautions that many interventions remain at an early stage, with recurring concerns around repetitive content, limited empathy, and sustained user engagement. Together, these findings suggest that while general-purpose chatbots can deliver meaningful gains, tinnitus-specific interventions that integrate CBT principles with sound-based and mindfulness elements are particularly effective in alleviating the unique burdens of tinnitus. They also underscore the potential of scalable, fully automated digital health tools for tinnitus care and the importance of addressing engagement and tailoring to clinical context. However, as only one general chatbot comparator was included, conclusions regarding general versus condition-specific interventions need to be interpreted cautiously.

This study has several limitations that should be acknowledged. First, as is common in online trials, participants self-selected and self-reported their tinnitus diagnosis, and no clinical or audiological assessments were conducted. All outcomes were based on self-report measures and were not corroborated by objective measures. While participants completed the TFI, a validated tinnitus-specific measure, future studies would benefit from incorporating clinical and audiological assessments to strengthen diagnostic confidence and outcome validity. In addition, detailed tinnitus characteristics such as laterality and perceived loudness were not collected, which may further inform individual differences in treatment response. Secondly, while engagement was encouraged through reminders, adherence data were not systematically collected. Future studies could address this by partnering with app developers to access usage logs and explore accurate adherence rates as a potential covariate of treatment response (Torous et al., 2020). Qualitative feedback and user experience data were not formally analysed within the present study, limiting insight into acceptability and participant engagement. Although qualitative data relating to user experience were collected as part of a related study, these were not analysed within the present manuscript, which focused on quantitative outcomes. Third, participant demographic information was limited, and variables such as educational background were not considered. This may influence the generalisability of findings to broader or more diverse populations. Follow-up was restricted to the eight-week intervention period to avoid delaying intervention access for the control group. While appropriate for this initial evaluation, future studies could incorporate longer-term follow-up to assess the durability of studied effects*.* In addition, participants were necessarily aware of receiving an app-based intervention, so blinding was not possible, and the possibility of a placebo effect is non-zero. The trial was not prospectively registered, which should be addressed in future confirmatory studies to strengthen transparency and reproducibility. Finally, multiple outcomes were assessed, raising the risk of Type I error. Although this reflects the multidimensional impact of tinnitus on mental health and wellbeing, future studies could gain advantage from larger samples and/or a more focused set of pre-registered primary outcomes.

Overall, the present findings extend the reach of internet interventions into tinnitus care, showing that app-based conversational agents can deliver transdiagnostic benefits and condition-specific advantages. By situating tinnitus within the broader field of digital mental health, this study underscores the promise of scalable, fully automated tools while also highlighting the importance of engagement, personalization, and rigorous evaluation. More generally, the study highlights tinnitus as a test case for how tailored conversational agents can be integrated into routine digital mental health practice.

## 5 Conclusions

App-based conversational agents delivering internet-based cognitive behavioural therapy content can reduce comorbid anxiety and depression in people with tinnitus. Here, only a tinnitus-specific application achieved a clinically meaningful reduction in tinnitus distress. These findings support the integration of scalable digital interventions into tinnitus care pathways. Tailoring interventions to tinnitus-specific examples offers added benefit beyond generic CBT approaches. Future trials should include usage analytics, longer follow-up, and prospective registration to test durability and support wider clinical adoption.

## CRediT authorship contribution statement

Conceptualisation: J.G.J.

Methodology: J.G.J., S.H., G.D.

Investigation: J.G.J. S.H., G.D.

Data curation: J.G.J.

Formal analysis: J.G.J., F.Y., B.M.

Writing - original draft: J.G.J. F.Y., B.M.

Writing - review & editing: J.G.J., F.Y., B.M., S.H., G.D.

Supervision: J.G.J.

Project administration: J.G.J.

All authors approved the final manuscript.

## Trial registration

This trial was not prospectively registered.

## Funding

This research was supported by a Bridging Grant from the Australian Academy of Technology and Engineering awarded to Dr. James Jackson (10.13039/100010050Leeds Trinity University). The funder had no role in study design, data collection, data analysis, data interpretation, or writing of the report.

## Declaration of competing interest

The authors declare no conflicts of interest.

## Data Availability

The data that support the findings of this study are available from the corresponding author upon reasonable request.
